# Exercise Training Prevents TNF-α Induced Loss of Force in the Diaphragm of Mice

**DOI:** 10.1371/journal.pone.0052274

**Published:** 2013-01-02

**Authors:** Norman Mangner, Axel Linke, Andreas Oberbach, Yvonne Kullnick, Stephan Gielen, Marcus Sandri, Robert Hoellriegel, Yasuharu Matsumoto, Gerhard Schuler, Volker Adams

**Affiliations:** 1 Heart Center Leipzig, University of Leipzig, Leipzig, Germany; 2 Department of Pediatric Surgery, University Hospital of Leipzig, Leipzig, Germany; 3 University Hospital - Martin Luther University of Halle/Wittenberg, Halle, Germany; 4 Department of Cardiovascular Medicine, Tohoku University Graduate School of Medicine, Sendai, Japan; University of Sydney, Australia

## Abstract

**Rationale:**

Inflammatory cytokines like tumor necrosis factor alpha (TNF-α) are elevated in congestive heart failure and are known to induce the production of reactive oxygen species as well as to deteriorate respiratory muscle function.

**Objectives:**

Given the antioxidative effects of exercise training, the aim of the present study was to investigate if exercise training is capable of preventing a TNF-α induced loss of diaphragmatic force in mice and, if so, to elucidate the potential underlying mechanisms.

**Methods:**

Prior to intraperitoneal injection of TNF-α or saline, C57Bl6 mice were assigned to four weeks of exercise training or sedentary behavior. Diaphragmatic force and power generation were determined in vitro. Expression/activity of radical scavenger enzymes, enzymes producing reactive oxygen species and marker of oxidative stress were measured in the diaphragm.

**Main Results:**

In sedentary animals, TNF-α reduced specific force development by 42% concomitant with a 2.6-fold increase in the amount of carbonylated α-actin and creatine kinase. Furthermore, TNF-α led to an increased NAD(P)H oxidase activity in both sedentary and exercised mice whereas xanthine oxidase activity and intramitochondrial ROS production was only enhanced in sedentary animals by TNF-α. Exercise training prevented the TNF-α induced force reduction and led to an enhanced mRNA expression and activity of glutathione peroxidase. Carbonylation of proteins, in particular of α-actin and creatine kinase, was diminished by exercise training.

**Conclusion:**

TNF-α reduces the force development in the diaphragm of mice. This effect is almost abolished by exercise training. This may be a result of reduced carbonylation of proteins due to the antioxidative properties of exercise training.

## Introduction

Impaired skeletal muscle function is frequently seen in a large variety of systemic disorders including congestive heart failure (CHF) and sepsis [1], [2]. While the above disorders develop in quite distinct clinical conditions, they all inflict similar skeletal muscles dysfunctions, including impaired contractility, reduced muscle protein synthesis, and enhanced muscle protein degradation and ubiquitination [1], [3], [4]. However, not only are the peripheral muscles affected by these mechanisms but also the respiratory muscles, in particular the diaphragm, which may also develop a secondary myopathy [5]. In the clinical context, respiratory muscle dysfunction is accompanied by increased morbidity and mortality in CHF [5], [6].

One important mechanism that is common in secondary myopathies is the prevalence of elevated inflammatory cytokines. Increased cytokine levels have been detected in the serum and/or in skeletal muscle tissues both in CHF and sepsis [7], [8], [9], [10]. Tumor necrosis factor alpha (TNF-α) is a proinflammatory cytokine that is elevated in the circulation of individuals with severe CHF promoting pathological symptoms [11], [12], [13]. Exogenous TNF-α administered either in vitro [14], [15] or in vivo [16], [17] depresses specific force (i.e., force per cross-sectional area) of limb and respiratory muscles. This response develops within hours [16], [17] and persists with prolonged exposure [18]. The decline in specific force is accompanied by an increase in muscle-derived oxidants and can be opposed by administration of antioxidants [18], [19].

Reactive Oxygen Species (ROS), e.g. hydrogen peroxide, are known to stimulate ubiquitin-conjugating activity and expression of genes for specific E2 and E3 proteins in skeletal muscle [20]. Recently, we were able to show that the TNF-α induced loss of muscle force is Muscle Ring Finger 1 (MurF1) dependent in peripheral soleus muscle, which therefore suggests the Ubiquitin-Proteasome-System (UPS) plays a central role in TNF-α induced force reduction [16]. The UPS is an ATP requiring multienzymatic process. Proteins degraded by the UPS are first conjugated to ubiquitin. This reaction requires the activation of ubiquitin by the ubiquitin activating enzyme (E1), transfer to an ubiquitin conjugating enzyme (E2), and subsequent linkage to the lysine residue of proteins destined for degradation (E3) [21].

It is well accepted that exercise training exerts antioxidative effects to the skeletal muscle in CHF, in particular due to an augmentation in the activity of radical scavenger enzymes [8], [22], [23], [24], [25]. Furthermore, exercise training reduces the catabolic activation caused by CHF in rats [26] and humans with moderate [27] and severe CHF [28].

The aim of the present study, therefore, was to test the hypothesis that an exercise training program is capable of preventing the TNF-α induced loss of muscle contractile force in the diaphragm of mice. In addition, potential molecular mechanisms responsible for the TNF-α induced force reduction and protective effects of exercise training were also analyzed.

## Materials and Methods

### Animal Groups

The local Council for Animal Research (Regierungspräsidium Leipzig/Saxonia/Germany) approved all experimental protocols (TVV 33/09). We performed a power analysis based on our results in soleus muscle prior to the study [16]. Choosing a power of 90% and 5% type I error, we calculated that a sample size of at least eight mice in each subgroup were required to detect significant differences (2 SD) in force development by ANOVA. With respect to possible animal loss during the study, a total of 40 female C57Bl6 mice (Medizinisch-Experimentelles Zentrum, University of Leipzig) were included. At the age of two months mice were randomized to exercise training (ET, n = 20) or a sedentary lifestyle (control group, n = 20). After a period of four weeks of ET or sedentary behavior, mice were randomly injected intraperitoneally (IP) with 100 ng/g body weight recombinant TNF-α (n = 10) (Sigma, Taufkirchen, Germany) or with an equal volume of physiological saline solution (n = 10). This dose of TNF-α is known to result in measurable and reliable effects of TNF-alpha on peripheral and diaphragmatic muscle function as described previously [16], [19]. The animals were sacrificed twenty-four hours after the injection of TNF-α using Pentobarbital (150 mg/kg body weight). Diaphragm bundles from the right hemidiaphragm were used for functional assessment by determining the force and power generation using a standardized in vitro electro stimulation protocol. The contralateral hemidiaphragm was frozen in liquid nitrogen for further analysis.

### Exercise Protocol

Mice assigned to the exercise group were accustomed to run on a motorized rodent treadmill with a shock-plate incentive. The slope of the treadmill was kept constant at 5°. Mice were trained at a speed of 15 m/min for 60 min/day with 2-minute rest intervals every 15 minutes for five days a week, as described previously [29]. All of the mice were confined to their individual cages throughout the study.

### Contractile Measurements

For functional assessment, a diaphragm bundle from the right hemidiaphragm was incubated in an oxygenated physiological buffer. The isolated muscle bundle was fixed with a silk suture at the central tendon and the proximal junction to the ribs, and mounted in an organ bath for measurement of muscle contractile function (Aurora Scientific, Aurora, Ontario, Canada) as described previously [16]. For a detailed description, please refer to Manuscript S1.

### Quantification of Enzymatic Activities

The frozen biopsy samples were homogenized in RIPA buffer containing proteinase inhibitors (Inhibitor mix M, Serva, Heidelberg, Germany) and protein content was determined (BCA assay, Pierce, Bonn, Germany). Enzymatic activities of NAD(P)H oxidase [30], aconitase [31], fumarase [32], glutathione peroxidase (GPX) [33], catalase [34], superoxide dismutase (SOD) and manganese superoxide dismutase (Mn-SOD) [35] as well as creatine kinase (CK) [36] were measured according to standard protocols and expressed as milliunits or units per milligram protein. Activity of xanthine oxidase was measured using a commercial kit according to the manufactures instruction (BioVision, Mountain View, CA, USA).

Calpain enzymatic activity was measured as recently described [37] using the fluorogenic peptide Suc-LLVY-AMC (Biomol, Hamburg, Germany) and a highly specific calpain inhibitor (Calpain inhibitor III, Calbiochem, Darmstadt, Germany).

### RNA Isolation and Quantification of mRNA Expression

Total RNA was isolated from diaphragm muscle tissue using RNeasy (Qiagen, Hilden, Germany), and reverse transcribed into cDNA using random hexamers and Sensiscript reverse transcriptase (Qiagen, Hilden, Germany). An aliquot of the cDNA was used for quantitative RT-PCR applying the LightCycler™ (Roche Diagnostics Inc). The expression of specific genes was normalized to the expression of Hypoxanthin-Phosphoribosyl-Transferase mRNA (HPRT). For a detailed description of the used primers and conditions, please refer to table 1 in Manuscript S1.

**Table 1:** Baseline characteristics of the mice and diaphragm strips.

	Sedentary+NaCl	Sedentary+TNF-α	Exercise+NaCl	Exercise+TNF-α
**body mass [g]**	18.3±0.8	18.8±0.7	18.9±0.9	18.0±0.7
**diaphragm strip length [cm]**	0.66±0.1	0.63±0.05	0.62±0.05	0.64±0.07
**diaphragm strip mass [mg]**	5.2±1.4	5.0±1.0	5.0±0.08	4.5±0.08
**diaphragm CSA [cm^2^]**	0.007±0.001	0.007±0.002	0.008±0.001	0.007±0.001

### Quantification of Protein Expression

Frozen tissue samples were homogenized in RIPA buffer containing a mixture of protease inhibitor (inhibitor mix M, Serva, Heidelberg, Germany) and protein expression was quantified by Western blot using specific antibodies to MuRF1 (generous gift of Dr. S. Labeit, University Mannheim, Germany) and MafBx (generated in rabbits against the following peptide sequence CYPRKEQYGDTLQL, Eurogentec, Seraing, Belgium). After incubation with a horseradish peroxidase-conjugated secondary antibody, specific bands were visualized by enzymatic chemiluminescence (Super Signal West Pico, Pierce, Bonn, Germany) and densitometry was quantified using a 1D scan software package (Scanalytics, Rockville, USA). Loading differences were controlled by re-probing the blot with an antibody against GAPDH (Hytest, Turku, Finland).

### Detection and Identification of Specific Carbonylated Proteins by 2D-gel Electrophoresis

Oxidized proteins were labeled with Alexa 488 Fluorescent Hydroxylamine and separated on a 2D-gel electrophoresis [38]. For further details, please refer to Manuscript S1.

### Proteasome Activity

The peptidase activities of the proteasome in the cytosolic fraction were determined as recently described [39]. For further details, please refer to Manuscript S1.

### Statistics

All data are expressed as mean value ± standard error of the mean. Intergroup comparisons were performed with two-way ANOVA followed by a post test according to Tukey using GraphPad Prism v.2.01, GraphPad Software, San Diego, CA. A probability value of <0.05 was considered statistically significant.

## Results

### Baseline

Animals of the four groups did not differ with regard to body mass, diaphragm strip lengths, diaphragm strip mass and diaphragm cross sectional area (table 1).

### Impact of TNF-α and Exercise Training on Force and Power Development in Diaphragm

**Figure 1:**
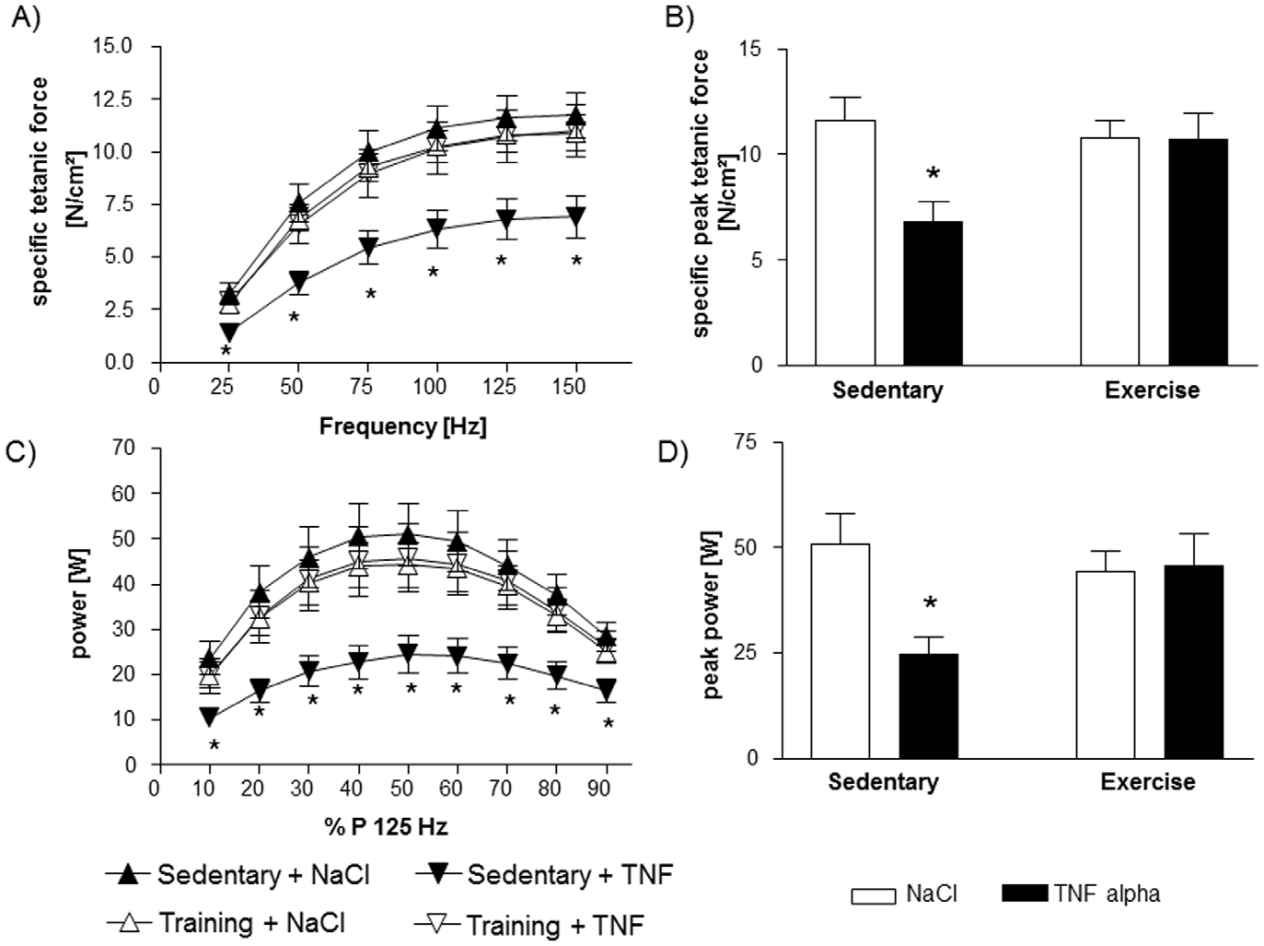
Force development is impaired in sedentary animals treated with TNF-α as shown by the force-frequency-relation (A). Specific peak tetanic force development (B) is reduced by ∼42% due to a single intraperitoneal TNF-α administration. This loss of force can be almost prevented by extensive exercise training four weeks prior to TNF-α administration. Power of the diaphragm is also reduced in sedentary mice by TNF-α, but this can be essentially prevented by exercise training (C and D). *p<0.01 vs. sedentary+NaCl and exercise+TNF-α; n = 10 per group.

As shown in Figure 1A, 24 hours after a single intraperitoneal injection of TNF-α into sedentary C57Bl6 mice, specific force development was significantly lower over the whole examined frequency spectrum in comparison to sedentary sham treated animals. Specific peak tetanic force development at 125 Hz was 42% lower in sedentary mice after administration of TNF-α (Figure 1B). As depicted in Figure 1A and B, exercise training almost entirely abolished the TNF-α induced loss of force in the diaphragm of mice.

Exercise training itself had no influence on the force development since there was no difference between exercised and sedentary sham treated mice.

Since power is calculated as the product of force (N/cm^2^) and shortening velocity (length/s) and no significant differences in shortening velocity were detectable (data not shown), power followed the same pattern as the above described force development: Figure 1C and D shows the diminished power in TNF-α treated sedentary animals compared to sham treated sedentary mice. Exercise training significantly attenuated the TNF-α induced power reduction.

### Expression and Activity of Antioxidative Enzymes

**Figure 2:**
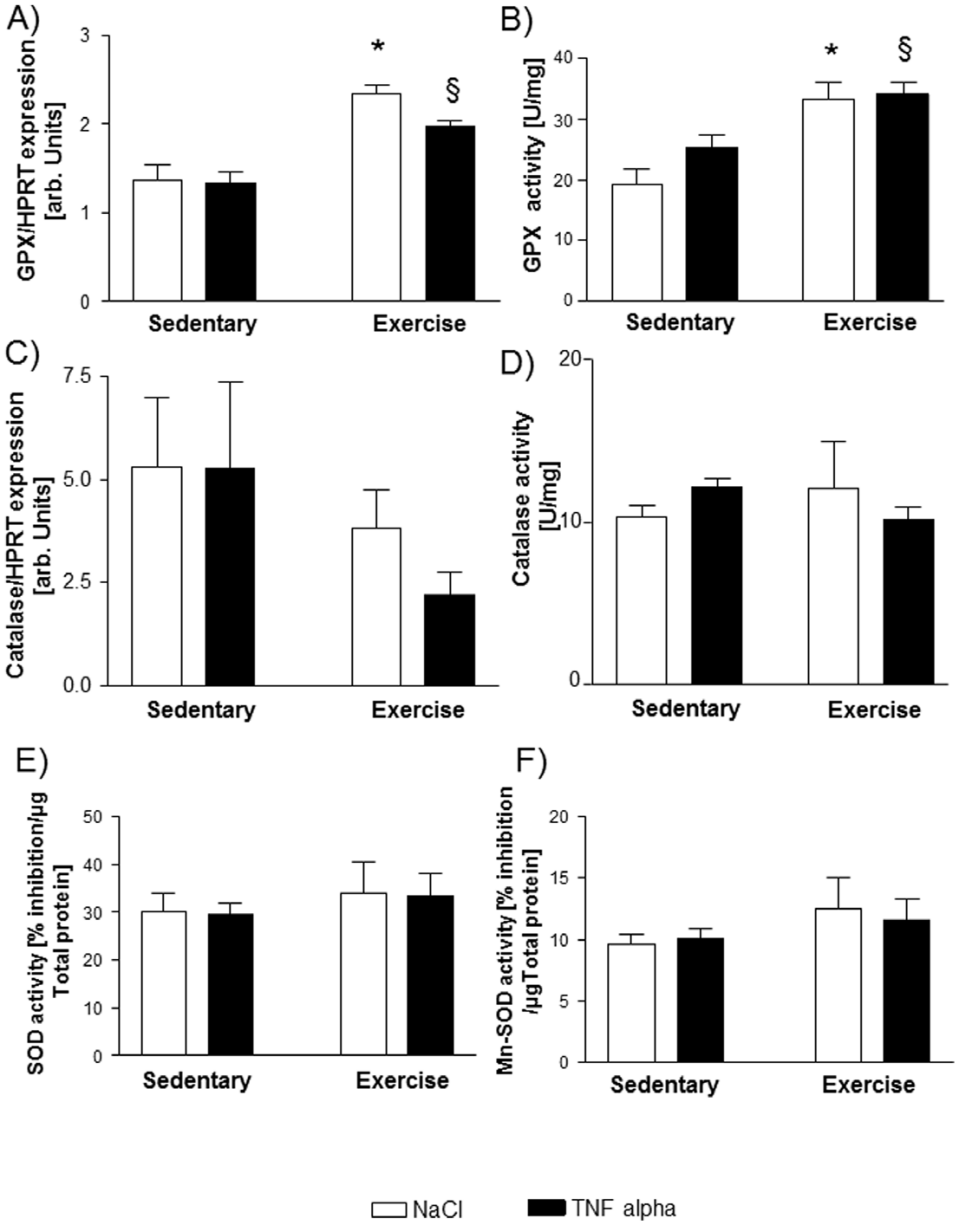
mRNA expression (A) and activity (B) of glutathione peroxidase (GPX) is induced by exercise training but not by TNF-α. Expression and activity of catalase is not influenced by exercise training or TNF-α administration (C; D). The activity of total superoxide dismutase (SOD) and manganese superoxide dismutase (Mn-SOD) is not affected by exercise training or TNF-α administration (E, F). *p<0.01 vs. sedentary+NaCl, §p<0.05 vs. sedentary+TNF-α, n = 10 per group.

As depicted in Figure 2A and B, mRNA expression and activity of GPX were significantly induced by exercise training. TNF-α itself had no influence on expression or activity of GPX. Neither expression nor activity of catalase was influenced by exercise training or TNF-α injection after 24 hours (Figure 2C and D). The activity of total SOD and Mn-SOD was also not altered by exercise training or TNF-α (Figure 2E and F).

### ROS Producing Enzymes and Markers of Oxidative Stress

**Figure 3:**
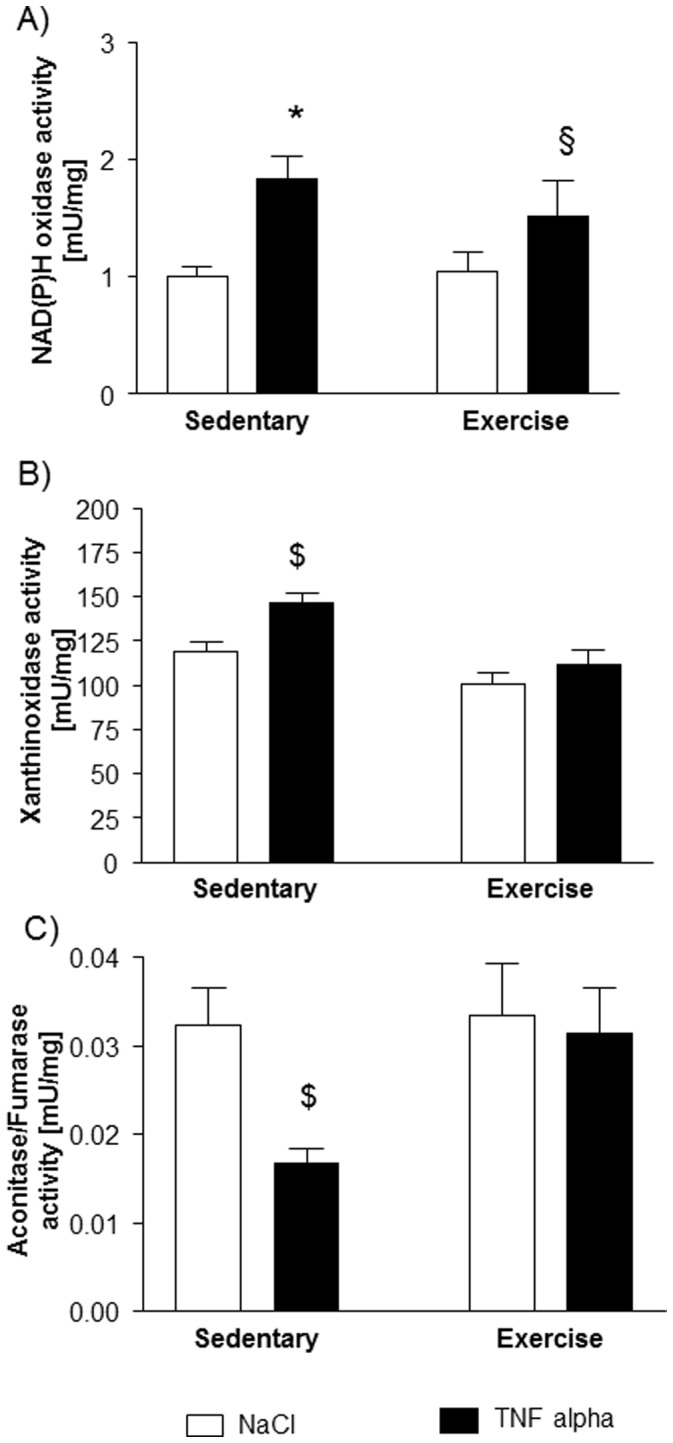
Possible sources of ROS: Activity of NAD(P)H oxidase (A) is induced by TNF-α in both sedentary and exercise trained animals. In contrast, activity of xanthine oxidase (B) and mitochondrial derived ROS, indirectly measured as the ratio of aconitase to fumarase activity (C), were only altered in sedentary mice treated with TNF-α whereas exercise training prevented an increase in xanthine oxidase activity or an inhibition of aconitase/fumarase activity via mitochondrial ROS by TNF-α. *p<0.05 vs. sedentary+NaCl, §p<0.05 vs. exercise+NaCl, $p<0.05 vs. sedentary+NaCl and exercise+TNF-α, n = 10 per group.

To determine the source of ROS, enzymatic activity of NAD(P)H oxidase was measured. After TNF-α administration, there was a significantly higher activity of NAD(P)H oxidase in both sedentary and exercised animals (Figure 3A).

Another important source of ROS, in particular the superoxide anion, is xanthine oxidase. As depicted in Figure 3B, activity of xanthine oxidase is enhanced in sedentary animals treated with TNF-α compared to sham ones. No significant increase in xanthine oxidase was detectable in exercised animals after the influence of TNF-α.

As an indirect marker of intramitochondrial ROS, the ratio of aconitase to fumarase activity was reduced in sedentary animals treated with TNF-α compared to shams indicating an increased intramitochondrial ROS concentration. No significant decrease in the ratio of aconitase to fumarase activity was detectable in exercised animals after TNF-α administration.

**Figure 4:**
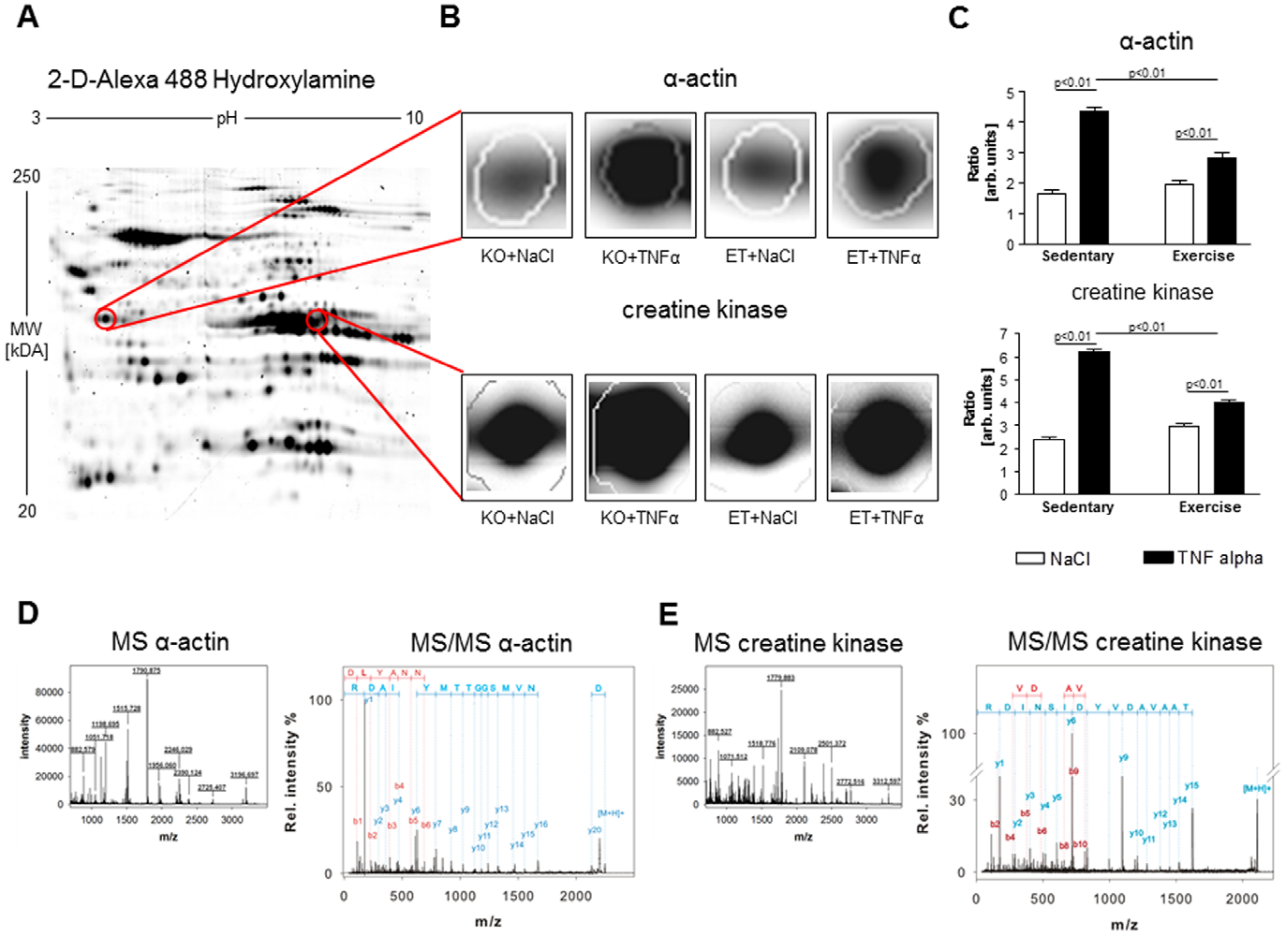
2-D gel electrophoresis (A) revealed 2.6-fold increase in carbonylated α-actin and carbonylated CK, depicted as a ratio of carbonylated α-actin to total α-actin or carbonylated CK to total CK, respectively. Exercise training diminished the carbonylation of α-actin and CK (B and C). In D and E the results of the MALDI TOF analyses are depicted confirming that the spots are α-actin and CK. KO = sedentary mice, ET = exercised mice. Sedentary+NaCl n = 7, Sedentary+TNF- α n = 8, Exercise+NaCl n = 8, Exercise+TNF- α n = 7.

Carbonylated proteins are an established marker of oxidative stress [40]. In search for specific carbonylated proteins which may be responsible for the TNF-α induced loss of force, we applied 2D-gel analysis specifically focusing on carbonylated proteins (Figure 4A). The most striking changes were seen in the myofibrillar protein α-actin and the energy providing enzyme creatine kinase (CK): We detected a 2.6-fold increase in carbonylated α-actin in sedentary animals after TNF-α treatment whereas only a 1.4-fold increase was evident in exercised animals (Figure 4B and C). Comparing sedentary animals treated with TNF-α with exercised animals treated with TNF-α, the amount of carbonylated α-actin was significantly lower in the exercise group (Figure 4B and C).

Furthermore, there was a 2.6-fold increase of carbonylated CK in sedentary animals after TNF-α treatment, whereas only a 1.3 fold increase was evident in exercised animals. Comparing sedentary animals treated with TNF-α with exercised animals treated with TNF-α again, the amount of carbonylated CK was significantly lower in the exercise group (Figure 4B and C).

**Figure 5:**
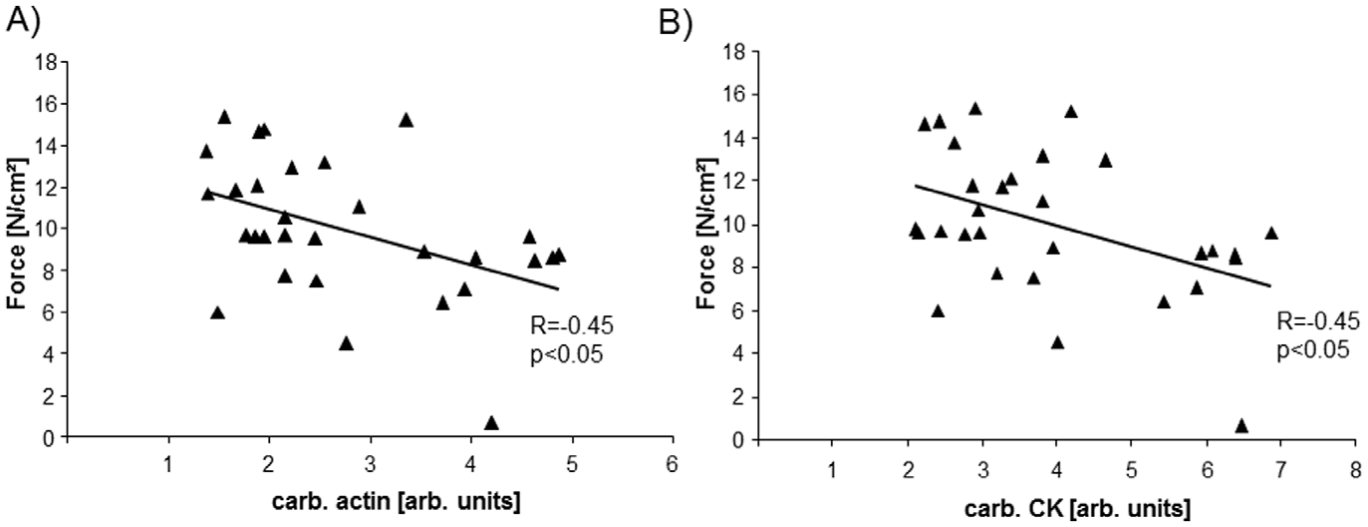
Carbonylation of α-actin (A) and CK (B), depicted as ratio of carbonylated protein to total protein, are inversely correlated to peak tetanic force development. Sedentary+NaCl n = 7, Sedentary+TNF- α n = 8, Exercise+NaCl n = 8, Exercise+TNF- α n = 7.

**Figure 6:**
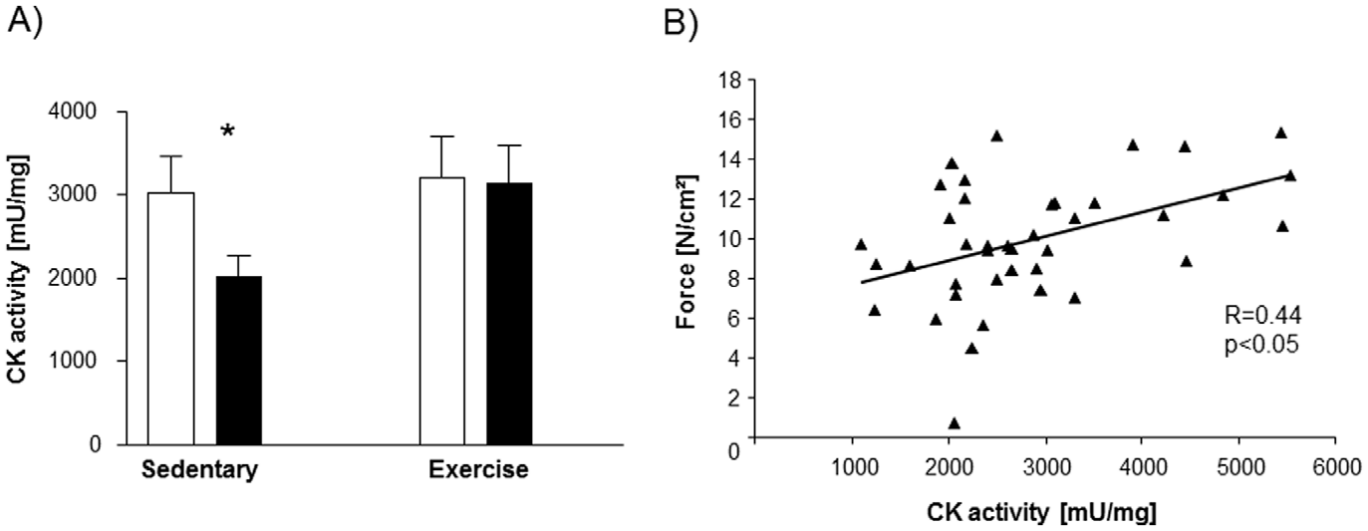
Total CK activity (A) in the diaphragm is reduced in sedentary animals treated with TNF-α compared to sham treated sedentary animals and TNF-α treated exercise trained animals. Activity of CK is correlated to peak tetanic force development (B). *p<0.05 vs. sedentary+NaCl and exercise+TNF-α; n = 10 per group.

Carbonylation of α-actin (Figure 5A) as well as CK (Figure 5B) was inversely correlated with force development. Furthermore, the activity of CK was significantly reduced in the diaphragm of sedentary animals treated with TNF-α, whereas no effect on CK activity after TNF-α administration was evident in exercised animals (Figure 6A). CK activity correlated significantly with force development (r = 0.44, p<0.05, Figure 6B).

### Expression of Ubiquitin E3-ligases, Proteasome Activity and Calpain Activity

Given that the muscle specific E3-ligase MurF1 plays a central role in TNF-α mediated loss of force in soleus muscle [16], we also examined the expression of MurF1 and a further E3-ligase MafBx in the diaphragm of our mice.

**Figure 7:**
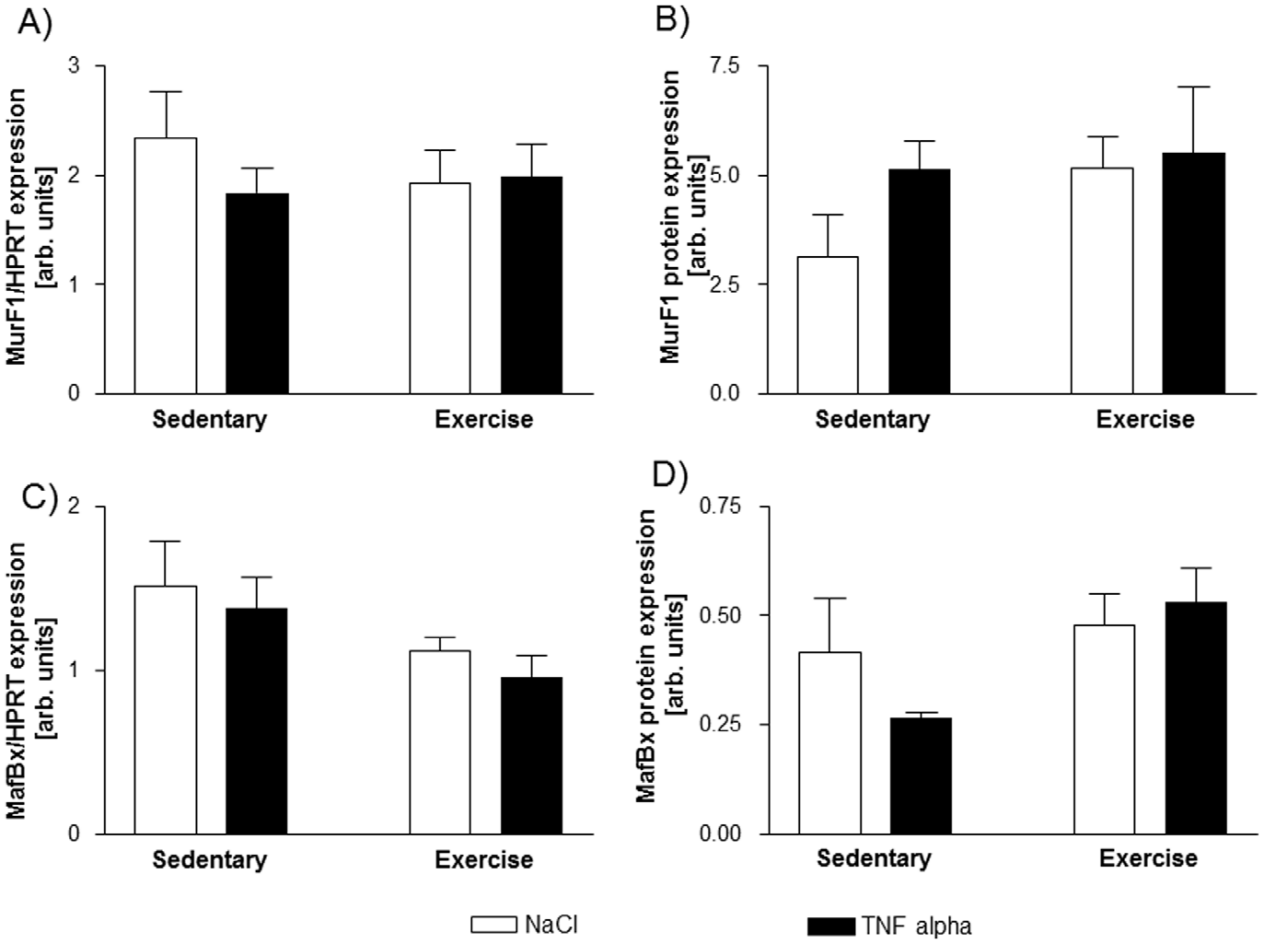
The mRNA and protein expression of MurF1 (A and B) and MafBx (C and D) is not affected by TNF-α or exercise training.

As shown in Figure 7, the mRNA and protein expression of MurF1 and MafBx were not altered by exercise or TNF-α administration in our animals.

**Figure 8:**
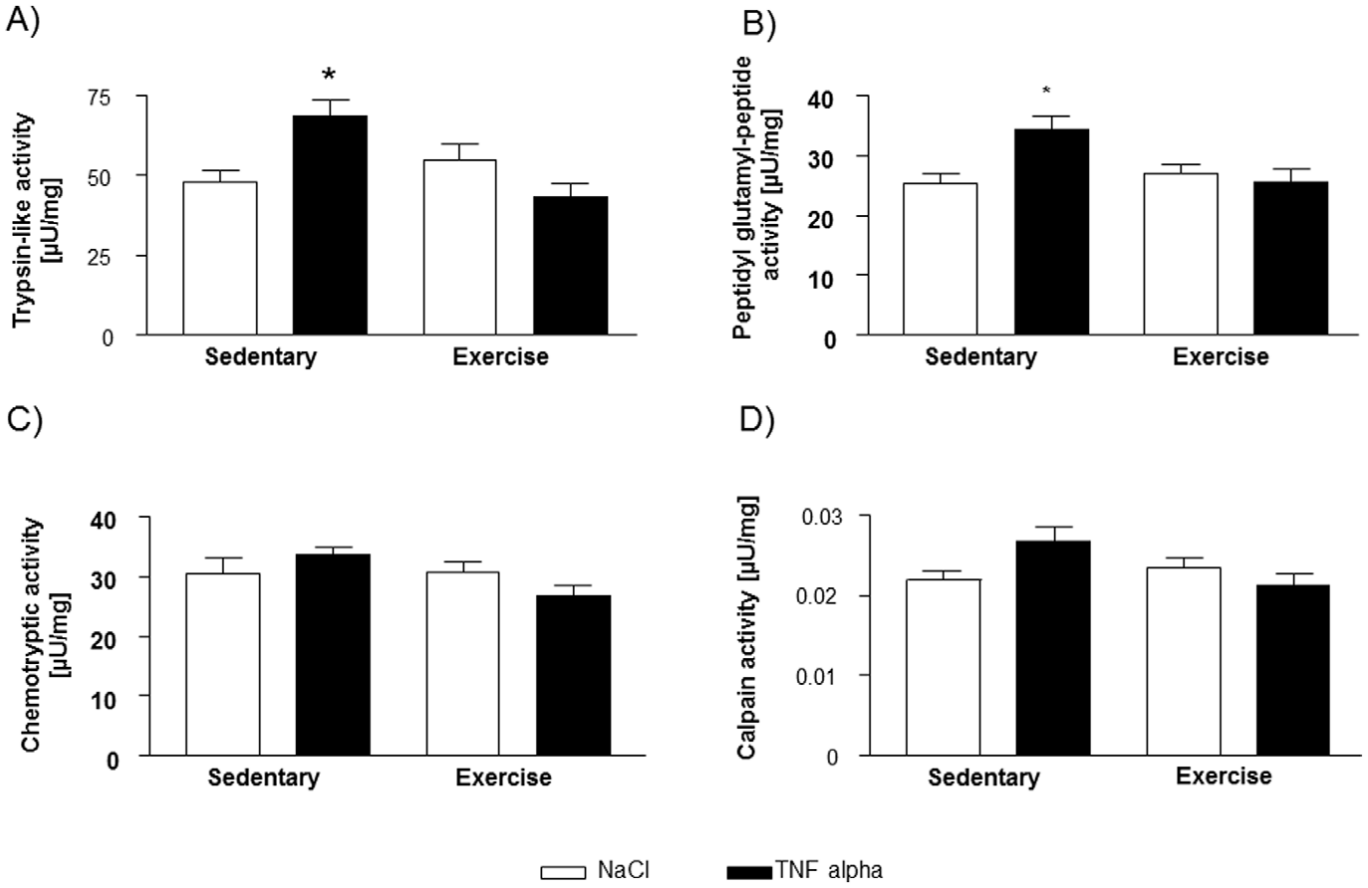
Trypsin-like (A) and peptidylglutamyl-hydrolizing activity of the proteasome (B) is enhanced by TNF-α in sedentary mice whereas no increase is detectable in exercise trained animals. Chemotryptic activity is not affected by both exercise and TNF-α (C). Neither TNF-α nor exercise training changed the activity of Calpain significantly (D). *p<0.05 vs. sedentary+NaCl and exercise+TNF-α, n = 10 per group.

The assessment of proteasome activity revealed a significantly higher activity under the influence of TNF-α in sedentary C57Bl6 mice, which was not detectable in exercised mice with respect to trypsin-like activity (Figure 8A) and peptidylglutamyl-peptide hydrolyzing activity (Figure 8B). Chemotryptic activity was not altered by a single intraperitoneal injection of TNF-α or exercise training (Figure 8C). Despite the enhanced proteasome activity, no difference in the total amount of main structural muscle proteins (α-actin, myosin light chain, troponin T, C and I) were detectable after this single and short term administration of TNF-α (see Figure S1). Noteworthy, the amount of carbonylated α-actin and CK were correlated to the above mentioned proteasome activities: R = 0.50 and R = 0.57 for α-actin with trypsin-like and peptidylglutamyl-peptide hydrolyzing activity, respectively (p<0.01) as well as R = 0.49 and R = 0.46 for CK with trypsin-like and peptidylglutamyl-peptide hydrolyzing activity, respectively (p<0.05).

Furthermore, calpain activity was not significantly different between the four groups in this experimental setting (Figure 8D).

## Discussion

Three key findings emerge from this study:

1. A single intraperitoneal injection of TNF-α induced a ∼42% decrease in specific force development in the diaphragm of sedentary mice after 24 hours. This loss of force was almost entirely prevented by intensive exercise training over four weeks prior to TNF-α administration.
2. TNF-α administration resulted in increased oxidative stress indicated by carbonylation of proteins, in particular of α-actin and creatine kinase. Exercise training was able to attenuate the TNF-α induced carbonylation of α-actin and CK. This may be due to an improvement in antioxidative capacity by the exercise program.
3. TNF-α treatment increased proteasome activity by ∼40% in the sedentary group. This increase was impeded by exercise training.

It is well accepted that TNF-α administration leads to impaired force development in the diaphragm of several animal models such as mice [19], dogs [17] and hamsters [41]. It is supposed that this effect is transmitted by reactive oxygen species since it is preventable by the administration of antioxidants such as Trolox [19].

However, exercise training also exerts antioxidative effects, in particular due to an augmentation in activity of radical scavenger enzymes [8], [23], [24], [25] mimicking the administration of an antioxidant. In this study, mRNA-expression and activity of glutathione peroxidase were enhanced by exercise training, whereas the enzymatic activity of catalase, SOD and Mn-SOD was not affected by the exercise intervention. This finding is in line with data derived from skeletal muscles of CHF patients, where the activity of GPX is enhanced by a six months exercise program but the activity of SOD is not [24]. In particular, the failure to increase superoxide anion producing enzymes in exercise trained animals (see below) may explain the unchanged activity of total SOD and Mn-SOD in those mice since they are the main detoxifying enzymes of superoxide anions.

NAD(P)H oxidase, one major source of ROS in skeletal muscle [40], showed an elevated activity in both sedentary and exercised animals after TNF-α injection. In contrast, however, intramitochondrial ROS (indirectly assessed by the ratio of aconitase/fumarase activity [42]) and the activity of xanthine oxidase (an important source of superoxide anions in skeletal muscle [40]) were only enhanced in sedentary animals treated with TNF-α, and exercise training prevented ROS production via xanthine oxidase or the mitochondria. Xanthine oxidase activity is induced by TNF-α and can be prevented by neutralizing anti-TNF-α antibodies [43]. The mechanism by which exercise training can prevent the TNF-α induced increase in xanthine oxidase activity remains unknown.

With regard to aconitase, ROS-mediated inactivation of the enzyme can be overcome, possibly by an exercise induced dephosphorylation of the enzyme [31]. These data indicate that exercise training affects both the production of ROS by TNF-α susceptible enzyme like xanthine oxidase and detoxification of ROS via increased glutathione peroxidase activity.

As mentioned above, ROS are supposed to play a central role in transmitting the force-impairment effect of TNF-α in the diaphragm [19], but it remains unclear which exactly defined proteins are affected by oxidation leading to force impairment. Protein carbonylation is a primary marker of oxidative stress, a state when ROS production increases to levels beyond the buffering capacity of muscle antioxidant systems [40]. Protein carbonylation is both irreversible and results in irreparable damage. It is a result of a particular type of redox reaction characterized by the addition of carbonyl groups and is the consequence of a cascade of several oxidative reactions [40]. Carbonylation of several myofilaments, mitochondrial and cytosolic proteins seem to contribute to cardiac and skeletal muscle dysfunction in both animal models of CHF, COPD, sepsis or cancer, and humans with eponymous diseases [44], [45].

For this reason, we searched for carbonylated proteins using 2D-gel electrophoresis. Due to the fast onset of diaphragm dysfunction, we assumed myofibrillar proteins and proteins involved in energy production as possible targets of carbonylation.

We detected a 2.6-fold increase in carbonylated α-actin and carbonylated CK in sedentary, TNF-α treated mice. This is in line with the current literature where α-actin and CK are described as the major proteins affected by carbonylation in many scenarios [40]. Exercise training attenuated this ROS mediated protein modification, indicating that carbonylation and consequently irreversible damage of the thin filament and energy transfer enzyme may be responsible for the TNF-α induced loss of muscle force in the diaphragm. This hypothesis is supported by an inverse correlation between carbonylated α-actin and CK with specific force development. Furthermore, we were able to detect a reduced total CK activity in sedentary animals treated with TNF-α. This TNF-α induced loss of activity was prevented by exercise training, with the activity of CK correlated to specific force development. Since the work of Bessmann and colleagues, it is generally accepted that an intact function of the CK shuttle is essential for muscle function [46].

The carbonylation of α-actin also has deleterious effects and destroys the organization of the thin filament. A study of Dalle-Donne and colleagues revealed that exposing actin to hypochlorous acid causes a rapid increase in carbonylation, a result of oxidation of methionines, tryptophans and lysines [47]. They also suggested that carbonylation of some methionine residues in actin results in strong inhibition of actin polymerization and a complete disruption of the actin-filament organization which may contribute to an impaired force development [47].

In contrast to the peripheral soleus muscle [16], neither MurF1 nor MafBx mRNA and protein expression were induced by TNF-α in the diaphragm of sedentary mice. Since there was a clear induction of MurF1 in peripheral soleus muscle, we assume that the ubiquitin dependent proteolytic function of the proteasome, contrary to the soleus muscle, does not contribute to the acute TNF-α induced loss of force in the diaphragm.

Interestingly, proteasome activity, in particular trypsin-like and petidylglutamyl-peptide hydrolyzing activity, was enhanced in sedentary animals by TNF-α which could be prevented by exercise training. Carbonylation of α-actin and CK was significantly correlated to the before mentioned proteasome activities. One possible explanation for this finding may be an ubiquitin-independent proteolytic function of the proteasome which is described for oxidized proteins [48], [49], linking carbonylation and proteasome system together. This linkage is supported by a study describing an accumulation of carbonylated proteins due to a diminished proteasome activity in astrocytes of chronic experimental autoimmune encephalitis mice [50].

Furthermore, a study by Supinski and colleagues provides strong evidence that the proteasome system itself is not directly involved in the acute force loss in the diaphragm and seems to be a bystander during this early phase of damage [51]. Testing three different proteasome inhibitors, none of these substances was able to restore the diaphragmatic loss of force due to administration of *E. coli lipopolysaccharide* despite a clear inhibition of proteasome based proteolysis [51]. Since the ubiquitin-proteasome system does not affect an intact protein lattice but only ones released from its original site, it is more likely that other proteases like caspase and calpain may play a role in the inflammatory diaphragmatic dysfunction. Indeed, Supinski and colleagues demonstrated that inhibition of both calpain and caspase was able to attenuate the diaphragmtic force loss induced by cecal ligation puncture, a model of sepsis [37]. In contrast, calpain showed no significant changes in activity under the single influence of TNF-α in sedentary and exercise trained animals in our study.

Since it was our aim to investigate the impact of one single inflammatory cytokine (TNF-α) on diaphragmatic function and to elucidate potential biochemical pathways, we used the artificial model of intraperitoneal injection of TNF-α. Therefore, the results of this study cannot be translated into certain diseases like CHF or sepsis. Further studies in animal models of CHF or sepsis are necessary to investigate the proposed mechanisms under the circumstances of a specific disease. In addition, we only investigated one specific exposure time of TNF-α (after 24 hours). For this reason, other mechanisms, e.g. enhanced proteolysis, may be relevant after long term exposure.

### Conclusion

**Figure 9:**
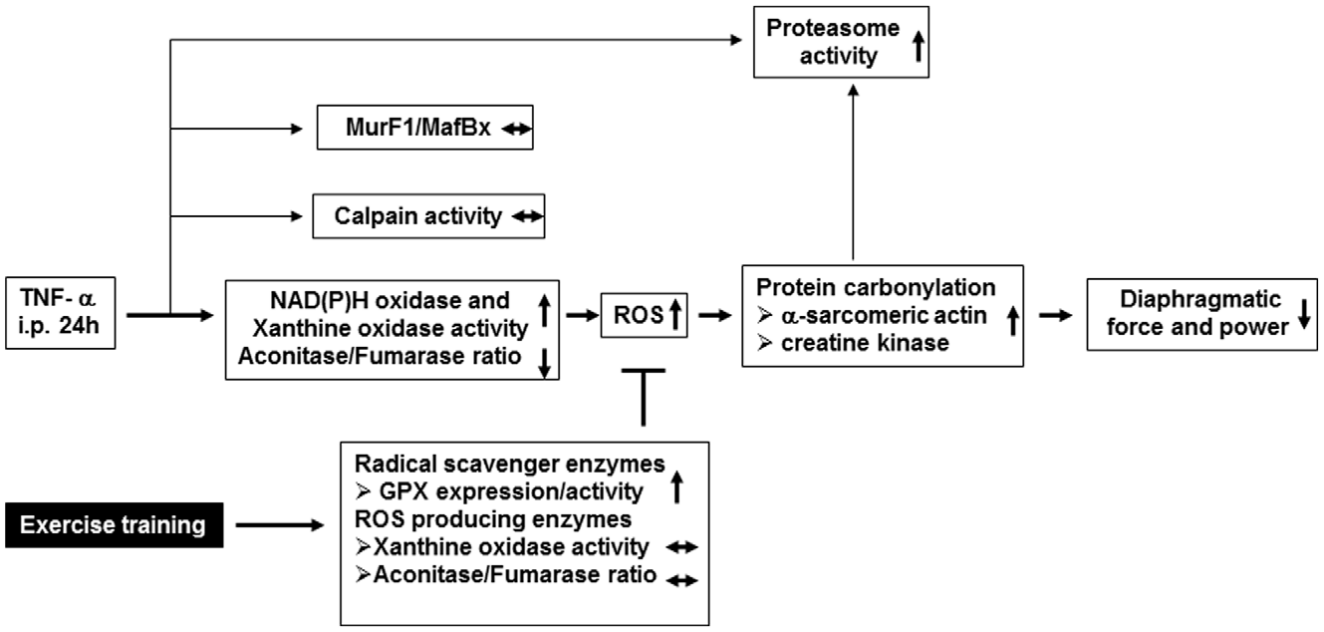
Working model for the TNF-α induced loss of contractile force in the diaphragm occurring after 24 hours and its prevention by exercise training. Thin lines indicate that these pathways are probably not involved in the TNF-α induced loss in diaphragmatic force as far as supported by our data.

In conclusion, the results of this study suggest that the administration of TNF-α led to a significant loss of diaphragmatic force and power. This was probably due to a ROS mediated increase in carbonylation of α-actin and CK, two important components of force generation. Exercise training, however, resulted in an elevation of the radical scavenger enzyme GPX as well as a prevention of TNF-α induced increase in xanthine oxidase activity and mitochondrial ROS production, thereby lowering the ROS mediated carbonylation of α-actin and CK to collectively preserve diaphragmatic force (Figure 9).

## Supporting Information

Figure S1 (TIF)

Manuscript S1 (DOC)
