# Supplementary figures and images for: Exercise Training Prevents TNF-α Induced Loss of Force in the Diaphragm of Mice
Source: PLoS One. 2013 Jan 2;8(1):e52274. doi: 10.1371/journal.pone.0052274 (PMC3534708; doi:10.1371/journal.pone.0052274)

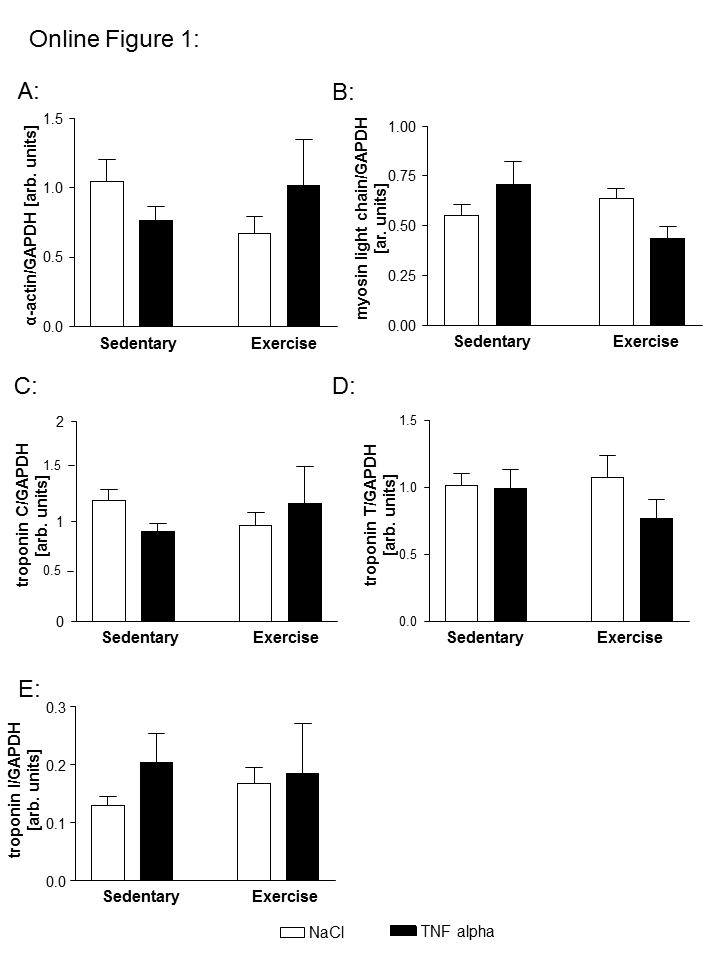

Supplement: Figure S1 — (TIF) [file pone.0052274.s001.tif]
