# Supplementary material for: Exercise Training Prevents TNF-α Induced Loss of Force in the Diaphragm of Mice
Source: PLoS One. 2013 Jan 2;8(1):e52274. doi: 10.1371/journal.pone.0052274 (PMC3534708; doi:10.1371/journal.pone.0052274)
Supplement: Manuscript S1 — (DOC) [file pone.0052274.s002.doc]

**Online Data Supplement**

**1. Methods**

**1.1 Contractile measurement**

For functional assessment, diaphragm bundles from the right hemidiaphragm were incubated in an oxygenated (95% O_2_ – 5% CO_2_) physiological buffer containing (in mmol/L) 120.5 NaCl, 4.8 KCl, 1.2 MgSO_4_, 1.2 NaH_2_PO_4_, 20.4 NaHCO_3_, 1.6 CaCl_2_, 10 dextrose, 1 pyruvate with a pH of 7.4 at 30°C. The isolated muscle bundle was fixed with a silk suture at the central tendon and the proximal junction to the ribs, and mounted in an organ bath for measurement of muscle contractile function (Aurora Scientific, Aurora, Ontario, Canada). Resting tension of the diaphragm bundle was continuously monitored and adjusted if necessary to 2.0 g throughout the measurement. Resting tension of 2.0 g was chosen based on a series of preliminary experiments in which resting tension of 2.0 g revealed the peak force development in both twitch and force frequency analysis representing optimal L_0_ of diaphragm bundles in our setting. In L_0_ the muscle was left quiescent for 15 min and was then subjected to a series of three isometric twitches (1 Hz) followed by three tetani (125 Hz) spaced 1 min apart to elicit consistent contractile responses before any additional experimental procedures were performed. After an additional 5-min quiescent period, the stretched muscle was subjected to a single isometric twitch (1 Hz) and a force-frequency protocol at electrical stimulation frequencies of 25, 50, 75, 100, 125 and 150 Hz, each for duration of 200 ms and with 1 min intermission.

Subsequently, the muscle was allowed to shorten after stimulation of 125 Hz (test duration 200 µs) while force was clamped at different levels ranging from 10 – 90 % of P at 125 Hz. The time window for shortening velocity measurements was set to begin 10 ms after the first detectable change in length. There was a 1-min interval between each force clamp level. The muscle shortening velocity at each load clamp was expressed as muscle lengths per second.

Results were adjusted to muscle cross-sectional area, which was calculated by dividing diaphragm strip weight (in g) by strip length (in cm) times specific density (1.056 g/cm³). Specific force per cross-sectional area was expressed as Newton per cm² (N/cm²). Power (force (N/cm²) x shortening velocity (length/s)) was calculated for each load clamp release and plotted with respect to % of force at 125Hz.

**1.2 Primer sequences for qRT-PCR analysis**

| **Gene** | **Sequence** | **Produduct**  **size** | **Annealing**  **temp.** |
| --- | --- | --- | --- |
| **Glutathione peroxidase** | AgTTCggACACCAggAgAATg |  |  |
|  | ATgTACTTggggTCggTCATg | 210 bp | 62°C |
| **Catalase** | gACAgTTCACAggTATCTgCA |  |  |
|  | AgACTCACCTgAAggATCCTg | 614 bp | 60°C |
| **Murf-1** | CCTTCACCTggTggCTATTC |  |  |
|  | gATgTgCAAggAACACgAA |  |  |
| FRET probes | gTggCATCgCCCAAAAgAACTTCATg |  |  |
|  | gCCTggTgAgCCCCAAACACCT | 259 bp | 55°C |
| **MafBx** | CAgACTggACTTCTCgACTgC |  |  |
|  | gAgTCTggAgAAgTTCCCgTAT |  |  |
| FRET probes | CTgATAgCAAAgTCACAgCTCACATCCCTg |  |  |
|  | gTggCATCgCCCAAAAgAACTTCATg | 202 bp | 55°C |
| **HPRT** | CTCATggACTgATTATggACAggAC |  |  |
|  | gCAggTCAgCAAAgAACTTATAgCC | 123 bp | 60°C |

**1.3 Detection of carbonylated proteins by 2D-gel electrophoresis**

*Sample Preparation:* Total protein was extracted from the diaphragm muscle using sample lysis buffer (7 M urea, 2 M Thiourea, 30 mM Tris, 4% (w/v) CHAPS, 1 mM PMSF and 1% protease inhibitor cocktail (Sigma-Aldrich, St. Louis, MO). Protein concentration was determined using a 2-D Quant protein assay kit (GE Healthcare, Uppsala, Sweden). Equal amounts of protein sample from the same treatment group were purified using a 2-Clean-up kit (GE Healthcare) for subsequent DIGE analysis.

For fluorescent detection of carbonyl, the oxidized proteins were labeled by adding 1 mg Alexa 488 Fluorescent Hydroxylamine (FHA, Invitrogen) to 20 mg of the oxidized proteins. The solution was allowed to react at room temperature for 2 h. 2DGE of the FHA-labeled proteins from whole muscle was performed as previously described [1] with the exception that experimental procedures were conducted in the dark. Briefly, FHA labeled proteins was precipitated with ice-cold trichloroacetic acid. The protein pellets were washed with ethyl acetate/ethanol (1:1) solution three times. Furthermore, the Alexa 488 Fluorescent Hydroxylamine labelled protein pellets from the diaphragm muscle were subsequent labelled with Cy3 Fluor minimal dye (GE Healthcare) according to the manufacturer’s recommended protocols. A total of 50 µg of protein from the treated and control groups were labeled with 400 pmol of Cy3. The labeled proteins (Alexa 488 Hydroxylamine + Cy3 labeling) were then adjusted to 450 ml with rehydration buffer (7 M urea, 2 M Thiourea, 2% CHAPS, 0.5% IPG buffer pH 4–7, and a trace of bromophenol blue) prior to isoelectric focusing (IEF) and subsequent SDS-PAGE.

*IEF and Gel electrophoresis:* Immobiline dry strips (pH 3-10, 24 cm, GE Healthcare) were rehydrated for 12 h in dark. The IPG strips were then focused using an Ettan IPGPhor 3 IEF system (GE Healthcare) as described previously [2]. In brief, the stripes were focused at 500 V for 1 h, 1000 V for 7 h, and at 8000 V for a total of 110 000 VhT at 20°C. Each gel strip was equilibrated twice, 15 minutes each time, with equilibration buffer (6 M urea, 30% glycerol, 2% SDS, 50 mM Tris-HCl, pH 8.8) containing first 1% w/v DTE, and then 2.5% w/v iodoacetamide and a pinch of bromophenol blue. The equilibrated strips were then carefully placed on top of 12% acrylamide gels and sealed with 1% w/v agarose. Second dimension separations were performed on an Ettan DALTtwelve electrophoresis system (GE Healthcare). The proteins were separated initially at 12°C, 1 W/gel for 12 hours, followed by 12 W/gel until the dye front reached the bottom of the gel. During all stages, work was carried out with great care in order to avoid its exposure to light.

Immediately after the run, DIGE gels were scanned within the gel cassettes using Ettan DIGE Imager Scanner (GE Healthcare). The Alexa 488 Hydroxylamine dye was excited at 488 nm and emission spectra were obtained at 520 nm, the Cy3 dye was excited at 532 nm and emission spectra were obtained at 580 nm The gels were visualized and first evaluated with the Image Quant Software, whereby all the three images as well as an overlay image of these images were checked individually. After scanning, the gels were removed from the gel cassette and were stained overnight with blue silver staining procedure. DIGE gel analysis was performed with Delta2D 4.0 software (Decodon, Greifswald, Germany) with advanced image processing algorithms that permits image fusion, background subtraction, normalization, and relative quantitation of proteins from different images. Following automated spot detection, each spot was manually verified and edited using 3D view algorithm. Ratio of Alexa 488 Hydroxylamine and Cy3 dye signaling of the same spots were identified after filtering the 0.5 to 2.0% ratio volume of the spots on the same gels. Furthermore, p-value was set at < 0.05 in all statistical group comparisons. Only these spots were given an identification number for further analysis.

*In-gel digests and sample preparation:* Protein spots of interest were cut from polyacrylamide gels and digested overnight using trypsin as described elsewhere [3]. The spots were washed two times with water: acetic acid: ethanol (50: 5: 45 v/v) for one hour. The gel spots were shrunk by the addition of acetonitrile, reduced and alkylated with DTT and Iodoacetamide. Afterwards, the gel slices were incubated with 5 mM ammonium bicarbonate, followed by incubation with acetonitrile 100% and dried by vacuum centrifugation. The dried gel pieces were swollen by adding trypsin in ammonium carbonate buffer (100 mM, pH 8.5) and were incubated at 37°C for 12 hours. Extractions of peptide were performed with extraction buffer containing acetonitrile: water: formic acid (5: 4.4: 0.6 v/v). The extracted peptide solutions were then completely dried using vacuum centrifugation. After that, the precipitates were reconstituted with 10 µl of 5% acetonitrile in 0.1% trifluoroacetic acid. 0.5 µl of this peptide extract and 0.5 µl of HCCA were mixed and spotted on ground steel target plates (Bruker Daltonics, Bremen, Germany).

*Protein identification by MALDI-MS/MS:* Peptide identification was carried out with MALDI TOF MS/MS (Ultra Flex III TOF/TOF, Bruker Daltonics, Bremen, Germany) using ground steel MTP 384 target plate (Bruker Daltonics) according to the manufacturer’s instructions. In brief, the samples were prepared by mixing equal volumes (1 µl) of sample and HCCA matrix (0.1 g/l of α-Cyano-4-hydroxycinnamic acid) and were then spotted onto the ground steel MTP 384 target plate. The solution was allowed to evaporate at room temperature. Spectra were obtained on an MALDI-TOF/TOF mass spectrometer (Ultraflex III™, using FlexControl software Vs. 3.0; Bruker Daltonics) as described earlier [3]. In brief, the instrument was operated at pulse rates of 100 Hz; pulse ion extraction delay was set to 400 ns as described earlier [3]. Measurements were carried out in positive reflector mode using an acceleration voltage of 25.0 kV (ion source 1) and 21.85 kV (ion source 2). Lens voltage was 9.5 kV, reflector voltages were 26.3 and 13.7 kV. Mass spectra were recorded in the m/z range between 200 and 3500. If possible up to 10 MS2 spectra were performed. The spectra were processed by the software FlexAnalysis (Bruker Daltonics). Thereafter a database search was conducted using the MS/MS ion search (MASCOT, Matrix Science, London, UK; version 2.2) against all metazoan (animals) entries of NCBInr (GenBank) with subsequent parameters: trypsin digestion, up to one missed cleavage, fixed modifications: carbamidomethyl (C) and with the following variable modifications: oxidation (M), peptide tol.: ± 1.2 Da, MS/MS tol.: ± 0.6 Da, peptide charge: +1, +2 and +3.

**1.4 Proteasome Activity**

The peptidase activities of the proteasome in the cytosolic fraction were determined as recently described [4]. Chymotrypsin-like, trypsin-like and peptidylglutamyl-peptide hydrolyzing activities were assayed using the fluorogenic peptides Suc-LLVY-AMC, Bz-VGR-AMC and Z-LLE-AMC, respectively (Biomol, Hamburg, Germany). 20 μg of cytosolic proteins were incubated with reaction buffer (0.05 mol/L Tris–HCl, pH 8.0, 0.5 mmol/L EDTA) and the respective labeled peptide (40 μmol/L). The kinetics of the reaction was recorded using a spectroflourimeter (Tecan safir^2^, Tecan, Crailsheim, Germany) with excitation at 380 nm (Ex) and emission at 440 nm (Em). Only the proportion of the reaction that could be inhibited by MG132 (20 μM, Sigma, Taufkirchen, Germany) was regarded as proteasomeal activity. For the calculation of enzymatic activity a calibration curve of free 7-amino-4-methylcoumarine (Sigma, Taufkirchen, Germany) was recorded. Values are expressed as microunits per milligram protein.

**2. Additional results**

**2.1 Expression of structural proteins**

Using quantitative western blot analysis no significant differences between the four groups were recognized with regard to the expression of major structural proteins (α-actin, troponin T, C and I (all antibodies by Santa Cruz Biotechnology, Heidelberg, Germany) as well as myosin light chain (antibody by Sigma, Taufkirchen, Germany) (online-figure 1).

**Figure Legend**

Online-Figure 1: Quantitative western blot analysis of α-actin (A), myosin light chain (B), troponin C (C), T (D) and I (E) revealing no significant difference between the four groups.

**References**

1. Poon HF, Castegna A, Farr SA, Thongboonkerd V, Lynn BC, et al. (2004) Quantitative proteomics analysis of specific protein expression and oxidative modification in aged senescence-accelerated-prone 8 mice brain. Neuroscience 126: 915-926.

2. Morbt N, Tomm J, Feltens R, Mogel I, Kalkhof S, et al. (2010) Chlorinated benzenes cause concomitantly oxidative stress and induction of apoptotic markers in lung epithelial cells (A549) at nonacute toxic concentrations. J Proteome Res 10: 363-378.

3. Jehmlich N, Schmidt F, Taubert M, Seifert J, von Bergen M, et al. (2009) Comparison of methods for simultaneous identification of bacterial species and determination of metabolic activity by protein-based stable isotope probing (Protein-SIP) experiments. Rapid Commun Mass Spectrom 23: 1871-1878.

4. Adams V, Linke A, Wisloff U, Doring C, Erbs S, et al. (2007) Myocardial expression of Murf-1 and MAFbx after induction of chronic heart failure: Effect on myocardial contractility. Cardiovasc Res 73: 120-129.
